# Advances in examination methods for adolescent idiopathic scoliosis

**DOI:** 10.1002/pdi3.2518

**Published:** 2025-01-09

**Authors:** Di Li, Peikang Wang, Man Zhang, Xinkai Zhang, Hailun Yao, Xing Liu

**Affiliations:** ^1^ Children's Hospital of Chongqing Medical University Chongqing China

**Keywords:** adolescent idiopathic scoliosis, diagnostic imaging, radiological examination, surface topography, ultrasound

## Abstract

The purpose of this article is to provide an overview of techniques for evaluating patients with adolescent idiopathic scoliosis (AIS). It encompasses the history, clinical examinations, and diagnostic imaging methods for AIS. These methods include digital radiological examination, EOS® imaging, nuclear medicine, ultrasound, body surface topography techniques such as the Moiré pattern technique, raster stereophotography, and DIERS formetric 4D as well as computed tomography and magnetic resonance imaging (MRI). Traditionally, full‐spine standing X‐rays have been the standard for diagnosing AIS. High‐quality clinical assessments may continue as a reference for assessing other spinal deformities. However, the new diagnostic imaging methods aim to reduce radiation exposure while maintaining image quality and practicality. Emerging technologies demonstrate strong reliability and effectiveness in diagnostic imaging of AlS. These techniques may be beneficial for diagnosing and monitoring AIS and its progression without requiring high levels of radiation exposure. The article is a search and summary of the PubMed electronic database to understand the current and future status of AIS imaging technology, which can not only help to introduce other researchers to the field but also serve as a valuable source for healthcare professionals to study existing methods, develop new ones, or select evaluation strategies.

## 1 INTRODUCTION

Scoliosis is a three‐dimensional structural deformity of the spine that includes abnormal alignment of the vertebrae in the coronal, sagittal, and horizontal planes. Depending on the cause, scoliosis can be categorized into several main types: idiopathic scoliosis (IS), congenital scoliosis (CS), neuromuscular scoliosis (NS), and heterogenic scoliosis. The most common type of scoliosis is idiopathic scoliosis (IS). 80% or more of idiopathic scoliosis occurs in adolescents and is called adolescent idiopathic scoliosis (AIS). According to the United States Preventive Services Task Force (USPSTF), the prevalence of AIS in children and adolescents aged 10–16 years is 1%–3%.^1^, ^2^ The natural history and risk of progression of idiopathic scoliosis in children and adolescents are determined by a number of factors, including skeletal maturity, gender, spinal curve type, and curve size, and idiopathic scoliosis is a structural scoliosis that develops gradually during growth and development.

The diagnosis of AIS usually bases on clinical symptoms and imaging. Typical symptoms include curvature of the spine, asymmetry of the shoulders and lower back, and tilting of the pelvis. A full‐spine standing X‐ray is a commonly used imaging modality to measure the angle of scoliosis and to determine the morphology of the spine. The degree of scoliosis varies from individual to individual, ranging from mild to severe. A scoliosis is clinically defined as a scoliosis with a scoliosis angle (the Cobb angle) of 10° or more.

Treatment of AIS includes observation, orthopedic treatment, physical therapy, and surgical correction. The choice of treatment usually depends on the patient's age, the degree and progression of scoliosis, and possible symptoms to ensure the most effective and individualized treatment plan. The main goals of treatment are multifaceted and aim to prevent the further progression of scoliosis and reduce the risk of long‐term pulmonary and cardiac sequelae that may result, as well as to correct the deformity of the spine and restore symmetry and balance to the trunk.^3^, ^4^, ^5^

**FIGURE 1:**
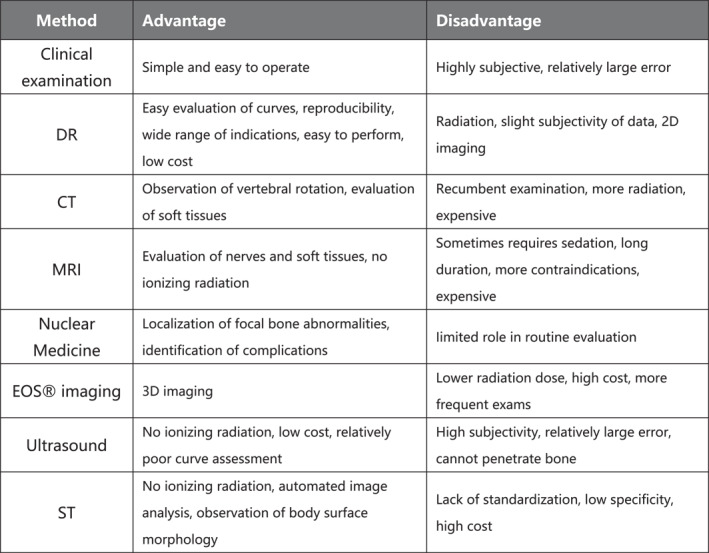
Advantages and disadvantages of imaging modalities mentioned in the text regarding AIS. AIS, adolescent idiopathic scoliosis.

The purpose of this article is to review the literature and discuss the role of the major imaging techniques in AIS. The development of each imaging modality is briefly discussed, and recent studies in the literature evaluating the role of these imaging modalities in pediatric idiopathic scoliosis are discussed. This article focuses on technical means and does not elaborate on issues related to physiotherapy and therapeutic methods. Each of these tests has its own advantages and disadvantages (Figure 1), and the gold‐standard status of the traditional method has yet to be shaken, despite its obvious drawbacks: a negative impact on the patient's health, subjectivity, and depletion of the physician. While emerging technologies aim to make the testing less risky and more convenient as technology develops, their reproducibility and reliability need to be further investigated, therefore leaving room for further improvement.

## 2 TRADITIONAL INSPECTION TECHNIQUES

### 2.1 Clinical examination

The idea that it is the child to be examined and not the scoliosis should be maintained. Record the patient's weight, height, and sitting height. Beginning to observe the child's posture as he or she walks helps to assess the overall posture. Then observe from the front, back, and side in a normal standing position. The pelvis should be level and lower limb discrepancies can be compensated for by lifting. A seated examination may be done in cases where the peripelvic situation is unclear (iliac spine torsion deformity, hip contracture, and sacroiliac joint pathology). A general neurologic examination is recommended; if any neurologic abnormality is suspected, a detailed examination is mandatory. Face the patient, and it is easy to observe asymmetries: head, shoulders, lumbar position, and alignment of the lower limbs. Observe skin pigmentation, facial deformities, and abnormalities of the teeth and palate. Assess chest mobility by observing chest expansion during deep breathing. Examine physiologic spinal curvature from the side. Sagittal distances from the plumb line to the back can be recorded at the cervical, thoracic, lumbar, and sacral levels,^7^ and alternatively curvature can be assessed using a pedometer. Asymmetries usually become apparent and are systematically recorded when viewed from behind the trunk: shoulders, scapulae, and lumbar region. Trunk balance is assessed by means of a plumb line placed from the tip of the C7 spine or the extraoccipital nodes. It is also important to check for symmetry in forward flexion of the back (Adams' test^7^). The trunk is gradually flexed forward and the observer can stop it at any angle and measure the trunk tilt with a scoliometer. That is, the angle of trunk inclination (ATI), also known as the angle of trunk rotation (ATR), is a parameter of high clinical value, comparable to the value of the radiographic Cobb angle.^6^ Although this method is simple and easy to perform, it is more subjective and has a relatively large error compared to imaging.

### 2.2 Digital radiography

Digital radiography (DR) remains the gold standard of imaging methods for scoliosis. Orthostatic and lateral standing radiographs are taken in normal (uncorrected) positions, and the magnitude of curvature is assessed by measuring the angle of scoliosis on the radiograph. As the simplest method, the Cobb method is widely accepted in the medical community. The earliest method was proposed by Ferguson in 1930, who assessed deformity by determining the angle between two straight lines connecting the centers of the terminal vertebrae to the centers of the parietal vertebrae, and by Cobb in 1948, who proposed another similar method for estimating the degree of scoliosis on radiographs.^8^ The method consists of positioning the most inclined vertebrae above and below the apex of the curve and measuring the angle between intersecting lines drawn vertically between the apical margin of the top vertebra and the bottom margin of the bottom vertebra.^9^ The Cobb angular measurement has become a quantitative criterion for recognizing and observing the symptoms of patients with scoliosis.^10^ In practice, it reflects only the inclination of the two limiting vertebrae and does not give any information about the length of the curve, vertebral rotation, or lateral shift of the apex. Spinal rotation is assessed by measuring the axial rotation of the vertebrae on orthopantomograms which is usually at the apical level. The method of evaluating the position of the pedicle shadow relative to the vertebral body shadow was proposed by Nash and Moe.^11^ Historically, lateral curvature of the spine was often analyzed by inclinometers, replicators, and even back cast models.^12^ The spine and the entire trunk undergo growth changes similar to torsion in response to muscles and ligaments.^13^

Cobb angles have utility in evaluating the initial curve, determining the magnitude of curve increase, and deciding when surgical intervention would be the most beneficial to the patient. The accuracy of Cobb angle measurement depends largely on the subjective experience of the radiologist. Previously, measurement was made using a device known as the Cobbometer, but the error was so great that it interfered with the diagnosis and treatment of patients with scoliosis. Therefore, in order to better evaluate full three‐dimensional spinal deformities with modern diagnostic imaging techniques, other methods of measuring the Cobb angle have been developed.^14^ Both the Cobb and Ferguson methods are based on manual identification of the end vertebrae. However, the Cobb method is preferred due to better reproducibility, easier application, and the ability to measure larger angles to assess more severe spinal curvatures. Today, the Cobb method has been standardized and the key aspect of “repeatability” has been tested and confirmed in many studies. By far the irreplaceable advantage of DR is the ability to calculate the angle of torsion and observe the morphologic changes in the vertebrae using the Cobb method.

### 2.3 Computed tomography

In idiopathic scoliosis, for reasons that are not yet clear, each vertebra in the curve is rotated from its normal position. This rotation can be characterized by the position of the longitudinal axis of rotation in which it occurs. Vertebral axis rotation has to a large extent been studied in vitro and in nonscoliosis. In these studies, the longitudinal axis of rotation was found to be determined by the orientation of the lesser joints and to be located predominantly within the confines of the vertebrae.^11^, ^15^, ^16^, ^17^ However, the location of the longitudinal axis of rotation of the spine as well as the different extra‐vertebral and intravertebral patterns of rotation in scoliotic spines, remains unknown, and the treatment and most of the etiologic concepts are based primarily on rotation.

Computed tomography (CT) scanning is considered the gold standard for the study of scoliosis spine rotation but there are limitations.^18^ First, CT scans are not performed upright but in the recumbent position. Previous studies have shown that both the Cobb angle and vertebral rotation are affected by body position.^19^, ^20^, ^21^, ^22^ The longitudinal axis of rotation may also vary between body positions. In contrast, on CT examination, the clinician must set sufficient parameters to better examine the extent of disease or scoliosis. Therefore, quantitative assessment of spinal curvature using specifically developed methods can improve medical diagnosis, treatment, and management of spinal disorders, and will support physicians in their work. The primary indications for spinal CT examinations include primarily the evaluation of congenital anomalies, alignment abnormalities, and traumatic injuries as well as postoperative evaluation, sometimes with intrathecal contrast.^23^ Enhancement of CT methods with three‐dimensional image processing is possible, which permits spatial imaging of the spine, detection of vertebral canal deformities, detection of congenital malformations of the spine, visualization of the position of spinal implants, and assessment of the quality of spinal immobilization.^24^ This examination plays an important role in the choice of surgical technique.

### 2.4 Magnetic resonance imaging

Magnetic resonance imaging (MRI) has revolutionized neuraxial imaging. Its ability to produce detailed images with excellent tissue contrast in any plane and without the use of ionizing radiation has made it an attractive method of evaluating scoliosis. However, the examination is very time consuming, and once the procedure has been performed, the presence of metalwork may make further MRI studies suboptimal, mainly because of magnetization artifacts from internal fixation. MRI is the technique of choice for patients with suspected spinal cord injury or compression in the presence of warning signs such as the cauda equina syndrome, tumors, or infections, or in the presence of complex low back pain (e.g. persisting for more than 6 weeks after conservative treatment).^23^ Also, young children require sedation and occasionally general anesthesia with MRI‐compatible equipment.

Nonetheless, MRI continues to be advocated as the primary imaging modality for scoliosis evaluation after radiographs,^25^ especially in infants and adolescents, where the incidence of spinal cord abnormalities is high. The role of MRI in adolescent scoliosis is unclear. Those children with atypical curves (e.g. left thoracic spine) or abnormal neurologic findings would benefit from MRI.^26^ MRI is used in the diagnosis of patients with scoliosis, primarily to assess neurologic structures and the shape of the spinal canal. Once the decision has been made to surgically correct scoliosis, some providers now routinely perform a preoperative MRI to obtain images of the entire spine, specifically including the craniocervical junction. Experienced radiologists can often simulate the curve in their own three‐dimensional mindset, a common misconception that scoliosis is simply a laterally deviated spine; however, the rotation of the associated vertebrae also plays a crucial role. Not only do the vertebrae rotate relatively to each other (axial intervertebral rotation), but there is also a degree of intrinsic rotation (axial intravertebral rotation or mechanical torsion), which can be accurately measured by CT,^27^ although this obviously exposes the child to further radiation. MRI may be beneficial to patients presumed to have idiopathic scoliosis, and its noninvasive and precise nature can help to accurately diagnose young patients with less unnecessary X‐ray exposure. Measurement methods have evolved since 2002, with Rogers et al.^28^ proposing a method based on measuring lumbar intervertebral rotation. This method has been used in MRI and CT.^29^ However, due to the high cost of MRI scans, they have been limited to studies of patients with congenital and severe curvatures.^30^

### 2.5 Nuclear medicine

Nuclear medicine has a limited role in the routine evaluation of scoliosis. Isotope bone scans (99mTc‐methylene diphosphonic acid) can be used in the initial evaluation of children to localize focal bone abnormalities such as infection or tumors, but are more commonly used in the postoperative spine to identify complications.

## 3 EMERGING SCREENING TECHNOLOGIES

### 3.1 EOS® imaging

EOS® (EOS® imaging, France) is essentially a low‐dose biplane radiography based on the pioneering work of Nobel Prize‐winning physicist Prof. Georges Charpak, who invented a gas particle detector with a multifilament orthogonal chamber.^31^ EOS® has the advantage of a low radiation dose compared to DR, the ability to reconstruct three‐dimensional images, and image the entire body, including the spine and lower extremities, in a functional standing position. The EOS® has been developed for orthopedic imaging with the ability to simultaneously capture high‐quality images in both posterior–anterior (PA) and lateral positions, which allows reconstruction of a three‐dimensional model, a device that can greatly enhance imaging of the spine. The EOS® allows for acquisition of images while the patient is in the upright weight‐bearing (standing, seated, or squatting) position, and can image the entire body. Images are captured while the patient is in an upright weight‐bearing (standing, sitting, or squatting) position, and the full length of the body can be imaged without the need to digitally stitch or manually join multiple images. The quality and nature of the images are similar to DR rather than CT. Since the advent of EOS® imaging, many studies have compared it to DR. The average skin radiation dose in the thoracoabdominal region is six to nine times lower with EOS® compared to DR.^32^ The average examination time is also significantly shorter with the EOS® system. In conditions such as spinal deformities in children, where repeated radiologic examinations are required throughout the child's growth years to monitor the progression of the deformity and make critical treatment decisions, these unique advantages of EOS® imaging place it in a very favorable position to become the imaging modality of choice for a wide range of spinal disorders. Nevertheless, it costs more and requires more radiology exams per year than DR. As such there is a need to find its balance in terms of applicability, diagnostic and treatment planning, radiation dose, price, and patient comfort.

### 3.2 Ultrasound

Modern 3D ultrasound imaging is becoming increasingly popular in the evaluation of structural changes in the spine because of its nonionizing nature and because it is performed upright and is inexpensive as well as user friendly. Suzuki et al.^33^ were the first to utilize ultrasound in the evaluation of spinal curvature and rotation. In more recent studies, different ultrasound markers have been identified and investigated for coronal plane spinal curvature assessment. The spinous process angle (SPA) is the angle formed between the lines drawn on the most inclined part of the spinous shadow. In these studies, SPA was used on coronal ultrasound images to assess coronal spine disease in patients with AIS, and the results were proved to be reliable and valid when compared with radiographic Cobb.^34^, ^35^, ^36^ A limitation of SPA is that it underestimates the severity of the curves when compared with conventional Cobb angle measurements,^34^, ^35^ and ultrasound cannot penetrate the bone, so only the posterior structures of the spine can be observed. With the development of hardware as well as reconstruction and rendering methods, real‐time 3D ultrasound imaging has become a promising technique in many clinical areas, including cardiology, surgical guidance, musculoskeletal tissue, and vascular imaging. However, to our knowledge, due to the complexity of spinal deformations involving intricate geometries and multilayers of soft tissues, there is currently no comprehensive study focusing on real‐time 3D ultrasound imaging for spine assessment. Recently, advances have been made in freehand 3D ultrasound that can visualize body anatomy in 3D space by combining conventional 1D array ultrasound probes with positional transducers, and a number of such systems have been developed for scoliosis assessment alone.^37^

### 3.3 Surface topography

Body surface topography (ST) is a photogrammetric technique that involves reconstructing the shape, size, and mutual position of objects based on photogrammetric images (photograms). It was developed by Drerup and Hierholzer in the 1980s.^38^ This radiation‐free technique projects horizontal streaks of light onto a participant's dorsal surface and records and digitizes a static image of these lines. Based on the deformation of the projected horizontal lines, a 3D image of the back surface can be generated, measured, and correlated with potential spinal curvature deformities.^39^, ^40^, ^41^ It has been successfully used to assess trunk deformities in children with scoliosis, where the relationship between spinal curvature angle and surface deformity has been in use. Recently, a novel scoliosis screening method was computed from a 3D virtual human model created by a 3D body‐fitting application and specific tights to assess scoliosis‐induced trunk asymmetry. Finally, the optimal threshold of *Z*‐values to detect moderate to severe scoliosis is determined by subject work characteristic curve analysis.^42^ The recognized advantages of ST include the noninvasive and safe nature of the examination, the ability to quickly and accurately assess body posture in three spatial planes, computerized data storage, and the acceptance of the examination by school‐aged children and adolescents. As a result, it is increasingly used in clinical practice.

However, the lack of standardization, low specificity, and higher hardware charges, for example, the need for specialized staff and separate rooms, cannot be ignored.

#### 3.3.1 The Moiré pattern technique

The projected Moiré technique, is a method of spatial photogrammetry (photographic topography), which deals with the reconstruction of shape and position, as well as the measurement of spatial objects based on so‐called photograms. Moiré stripes are an arrangement of stripes that result from the interference (overlap) of two gridlines rotated by a certain angle or deformed.^43^ The development of the Moiré method has taken place in two directions: the first is the replacement of the traditional grating by an optical grating, which is a positive slice of the stripes that are projected onto a surface from a slide projector. The second direction is related to the development of modern computer technology. The general principle of obtaining information about the shape of the surface using Moore's technique is based on the analysis of the image of a linear grid (grating) displaced by optical means onto the examined surface. There is a characteristic difference between the contour lines of the two halves of the body in patients with scoliosis. Currently, the aim of this technique is to simplify and automate the measurement method.^8^

#### 3.3.2 Raster topography with automatic image analysis

Advances in computer technology have led to the development of raster topography methods with automated image analysis (also known as raster stereophotography).^44^ Raster topography evaluates 3D spinal deformities by analyzing the dorsal surface topography based on the principle of triangulation.^45^ The traditional raster was replaced by an optical grating, which is a positive slice of the stripes projected by a slide projector onto the surface being measured. A special optical system with a camera captures the image and transmits it to a computer. The computer detects any distortion of the raster lines and, based on the automatically detected anatomical fixation points, which can be calculated on the basis of the specific convexity of the spinous processes of the vertebral protrusions and the prominence of the lumbar fossa as a point of fixation,^45^ reconstructs a three‐dimensional model of the spine using mathematical algorithms, automatically analyzes it, and generates a morphometric map of the patient's body surface.^8^

#### 3.3.3 DIERS formetric 4D

Due to its noninvasive, noncontact, and radiation‐free ability to observe posture and spinal deformities, a surface morphology instrument, the DIERS formetric 4D (DIERS Medical Systems, Chicago, IL, USA), allows researchers to observe the full contours of postural changes without the dangers associated with radiography. Using the DIERS formetric 4D, a typical scan of the back for static standing posture analysis takes 6 s.^43^ The device used for the three‐dimensional examination consists of two main components. The first is a digital camera and the second is a projector. The device emits a measuring beam and directs it to the patient's spine. The data collected in this way is immediately transferred to a computer and a special program creates a digital map of the specified body part. The obtained measurements can be used at a later stage to diagnose the problem and indicate the appropriate course of action in case of disease. During the scanning process, shape parameters such as angles, distances, rotations, and deviation calculations of the spine and pelvis are collected. To determine the individual shape parameters reported from a series of images, the algorithm calculates the average of the entire scan from a specific parameter. As a method of data reduction, the algorithm selects the one of the images that is closest to the average and reports the value of the spine shape parameter for that image. And it excludes the most common measurement errors, known as human factors. Because of its availability, it is an ideal alternative to invasive examinations and is perfectly suited to assessing results before and immediately after treatment.^39^ The examination is characterized by a high degree of accuracy and, because of the ability to view the examined area directly, the physician can analyze it realistically and in real time.^43^ The three‐dimensional analysis of the spine can also be used in pregnant women.^8^

## 4 CONCLUSION

After briefly describing the techniques used to assess patients with scoliosis, we discovered that the methods for assessing spinal curvature are continuously evolving, with many scientists working to develop fully computerized methods for quantifying curvature. Despite the availability of advanced tools, we encountered a significant problem: the reproducibility of results is severely lacking. Different statistical methods being used make it challenging to compare results effectively.

Therefore, in the author's opinion, there are advantages and disadvantages to each of the several examination modalities, and more accurate radiography, or CT, etc. can be used for testing at critical points where a clear diagnosis or indication for surgery is needed, and where the patient's next diagnostic and treatment plan is being developed. As for the new technology, it can be applied to the screening of scoliosis, patient follow‐up monitoring, and evaluation.

## AUTHOR CONTRIBUTIONS

Di Li organized the data, designed and researched the database, performed the data analysis, and produced the first draft of the manuscript. Peikang Wang and Man Zhang contributed to the drafting and revision of the manuscript. Xinkai Zhang was involved in the conception and design of the study. Hailun Yao contributed to the content analysis. Xing Liu is the guarantor of the article. He took full responsibility for the work and conduct of the study, had access to the data, and supervised publication decisions. All authors have read and approved the final submitted manuscript.

## CONFLICT OF INTEREST STATEMENT

There are no conflicts of interest in this article.

## ETHICS STATEMENT

This study is a review and did not involve clinical data and human specimens.

## Data Availability

Data sharing not applicable to this article as no datasets were generated or analyzed during the current study.
